# Restorative effects of urban biodiversity: a study based on age differences using multigroup structural equation modeling

**DOI:** 10.3389/fpsyg.2026.1907289

**Published:** 2026-08-28

**Authors:** Rong Chen, Pei-ying Weng, Lvlin Miao, Zheng Lin, Xia Zhao

**Affiliations:** 1 School of Art and Design, Fuzhou Technology and Business University, Fuzhou, China; 2 College of Life Sciences, Fujian Agriculture and Forestry University, Fuzhou, China; 3 Ningde Urban and Rural Planning Institute, Ningde, China; 4 Fujian Rongsheng Construction Development Co., Ltd., Fuzhou, China

**Keywords:** ageing, attention restoration theory, biophilic design, multi-group structural equation modelling, perceived biodiversity, perceived restorativeness, positive affect, urban green infrastructure

## Abstract

Rapid urbanisation has exacerbated public mental health burdens, and contact with urban green spaces is widely promoted as a low-cost restorative countermeasure. Yet the mechanistic pathways through which multisensory nature perception confers psychological benefit — and whether these pathways vary systematically across the human lifespan — remain poorly characterised. Here we report a quasi-experimental interventional field study conducted along three ecologically stratified walking trails in a large urban park in Fuzhou, China (*N* = 360; young adults, 18–35 yr.; middle-aged adults, 36–59 yr.; older adults, ≥60 yr). Using multi-group structural equation modelling (MG-SEM) restricted to the natural trail subsample (*n* = 240), we show that nature trails significantly elevated positive affect (*p* < 0.001), with visual plant complexity and birdsong emerging as the primary correlates of perceived restorativeness. Crucially, the indirect association linking perceived biodiversity to positive affect via PRS exhibited pronounced age-cohort variations, becoming prominent and highly significant specifically within the older adult cohort: young adults β = 0.052 (*p* = 0.138); middle-aged adults β = 0.219 (*p* = 0.051); older adults β = 0.574 (*p* < 0.001). These findings suggest that older individuals are more profoundly dependent on high-quality multisensory nature experiences to sustain psychological well-being — a pattern consistent with the cognitive resource depletion hypothesis of ageing. Our results provide actionable evidence for designing age-inclusive therapeutic landscapes and biodiversity-informed urban green infrastructure.

## 1 Introduction

Accelerating urbanisation has fundamentally reshaped the physical and social environments in which most of humanity now lives, simultaneously amplifying exposure to chronic stressors — including noise, air pollution, social density, and the near-total displacement of natural elements — that collectively underpin escalating rates of anxiety, depression, and cognitive fatigue. Urban forests and associated nature-based interventions have emerged as a compelling solution to this challenge, with a growing corpus of evidence confirming their capacity to provide cultural ecosystem services that buffer environmental stress and promote psychological health (Berman et al., 2008). Urban green spaces (UGS) and associated nature-based interventions have emerged as a compelling solution to this challenge, with a growing corpus of evidence confirming their capacity to buffer environmental stress and promote psychological health. The theoretical basis for this body of work is deeply rooted in environmental psychology frameworks: Stress Recovery Theory (SRT) (Ulrich et al., 1991), which suggests that passive exposure to natural environments leads to quick physiological and psychological recovery mediated by affect; and Attention Restoration Theory (ART) (Kaplan, 1995), which asserts that natural settings evoke involuntary fascination instead of directed attention, thereby facilitating the replenishment of exhausted cognitive resources. Despite this theoretical coherence, identifying precisely which environmental features trigger these restorative cascades remains a central and unresolved question (Olszewska-Guizzo et al., 2022).

While previous research has separately established the restorative potential of vegetative richness and avian vocalizations (Wood et al., 2018; Qi et al., 2022), our study addresses a significant empirical lacuna by examining their coordinated interplay within a multisensory-lifespan integration framework (Korpilo et al., 2024). Rather than a simple additive combination, we propose that the combined visuoauditory effects of visual plant complexity and characteristic natural soundscapes (e.g., birdsong) enhance environmental fascination and psychological restoration (Qi et al., 2022; Korpilo et al., 2024). Furthermore, moving beyond the “sample monoculture” of young undergraduates prevalent in seminal models—such as the framework by Nghiem et al. (2021)—this study provides incremental value by systematically testing variations across distinct age cohorts (Yu et al., 2020). This approach refines Attention Restoration Theory (ART) by demonstrating how age-related cognitive resource depletion alters environmental dependency (Zhang et al., 2025).

A further limitation pervades the existing mechanistic literature. Seminal models — including the influential framework of Nghiem et al. (2021) — have been constructed almost exclusively from convenience samples of homogeneous young undergraduates. This sample monoculture obscures potentially consequential heterogeneity in how individuals at different life stages perceive and respond to natural environments. As global populations age, the natural attrition of cognitive resources that accompanies ageing may fundamentally alter an individual’s sensitivity to restorative environmental cues. Recent studies in the “age × restorativeness” domain suggest that high-quality, species-rich environments confer the greatest psychological benefits to older adults, who may rely more heavily on external environmental quality to compensate for internal cognitive resource depletion (Yu et al., 2020; Zhang et al., 2025). Building on this, our study systematically examines whether the mediating role of perceived restorativeness significant variations across distinct age groups (Uebel et al., 2021).

**Figure 1:**
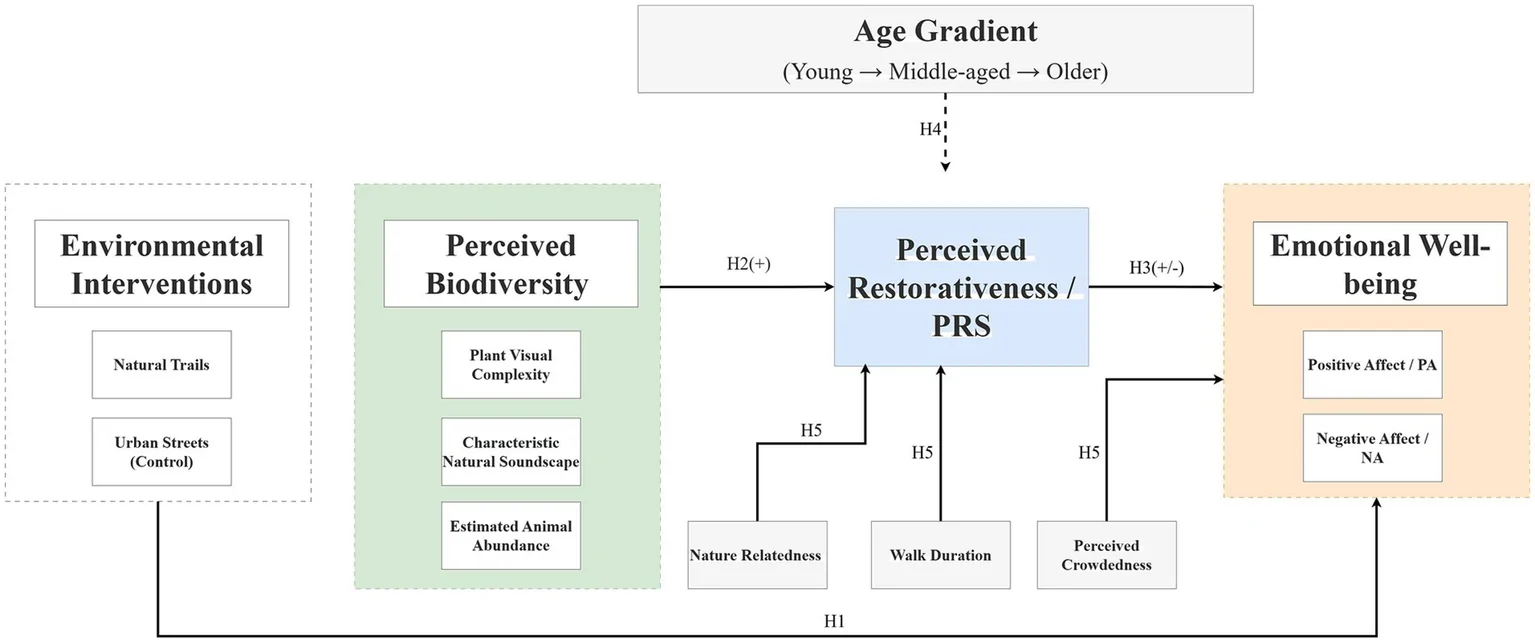
Conceptual model illustrating the hypothesised pathways linking multisensory perceived biodiversity to affective well-being, mediated by perceived restorativeness, with age cohort as a moderating variable.

More fundamentally, these emerging research gaps underscore the need for a comprehensive multisensory-lifespan framework to assess how integrated visual–auditory environments function as restorative resources across diverse age groups. By consolidating the previously parallel questions of biodiversity perception, cross-modal integration, and age-related sensitivity, this study moves beyond simple additive models. We investigate how the coordinated interplay of visual and auditory stimuli (e.g., birdsong) triggers restorative cascades, and whether this dependence intensifies as a function of age-related cognitive decline. Under this unifying theoretical throughline, Hypotheses 1–3 serve as necessary preconditions to validate the multisensory effect, while Hypothesis 4 tests the core distinctive contribution of our age-differentiated framework (Figure 1).

*Hypothesis 1* (H1 — Environmental main effect): Relative to the zero-biodiversity urban hardscape control, walking along nature trails will produce significantly greater increases in positive affect and decreases in negative affect.

*Hypothesis 2* (H2—Visuoauditory antecedents) states that multisensory perceived biodiversity, which is here operationally defined as visual plant complexity and characteristic natural soundscapes (e.g., birdsong), will reliably and positively predict perceived restorativeness.

*Hypothesis 3* (H3 — Restorative mediation): Perceived restorativeness will function as a significant full or partial mediator between perceived biodiversity and post-walk affective outcomes.

*Hypothesis 4* (H4 — Age-group variations): The indirect association linking perceived biodiversity through perceived restorativeness to affective well-being will exhibit significant differences across the three age cohorts, with older adults showing a stronger and more prominent association than middle-aged and young adults (Yu et al., 2020).

*Hypothesis 5* (H5 — Covariate-specific effects): Nature relatedness will positively predict perceived restorativeness. Furthermore, actual walking duration and perceived crowding are specified as covariates, where perceived crowding is hypothesized to negatively associate with perceived restorativeness.

## 2 Materials and methods

### 2.1 Study area and environmental typology

**Figure 2:**
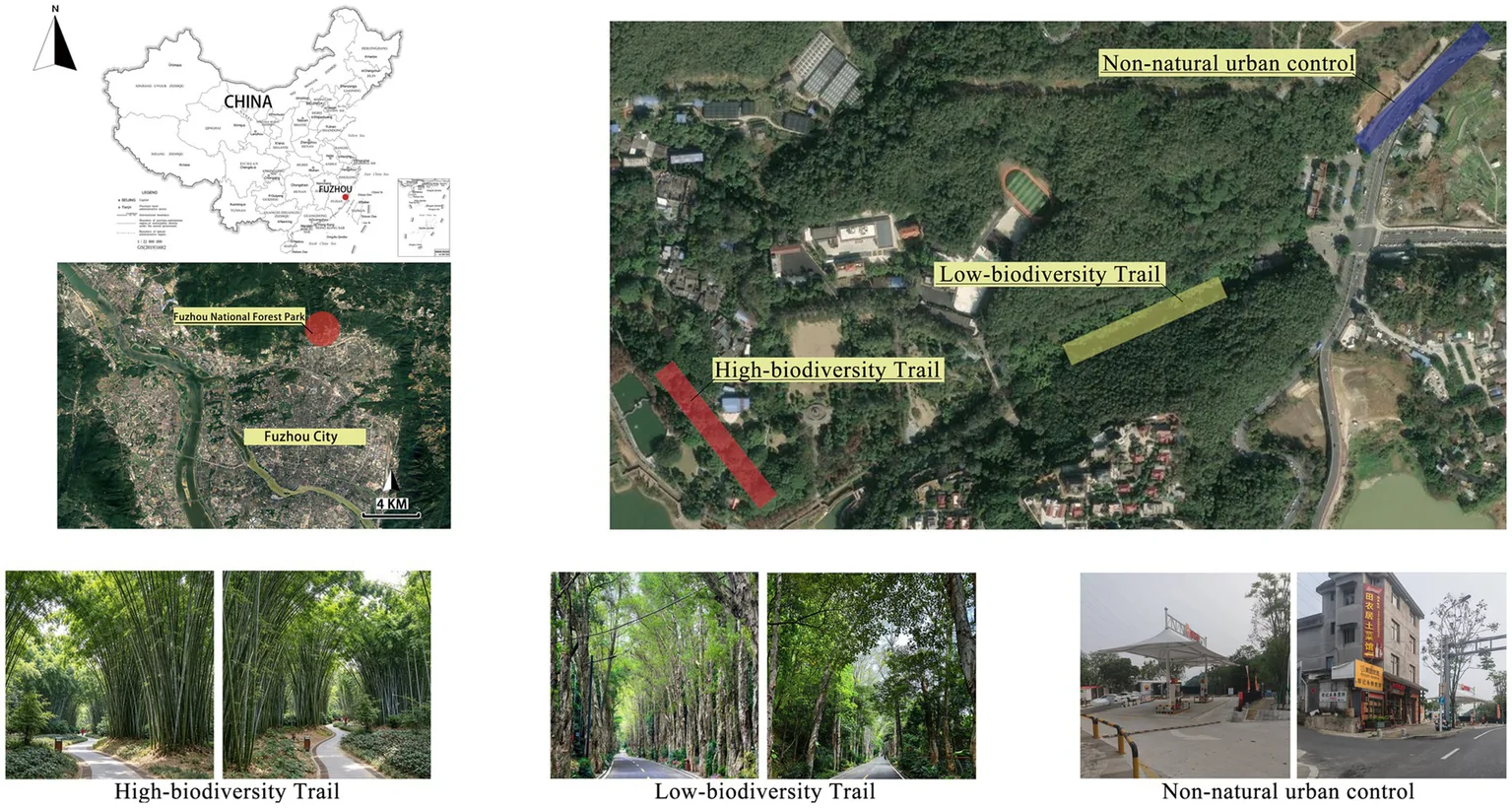
Map of the study area with a zoomed-in inset showing the spatial distribution of the three independent walking trails, and representative scenes of the environmental typologies. Left: High-biodiversity Trail (HFP); centre: Low-biodiversity Trail (MIP); right: Non-natural Urban Control (UHS).

The study was carried out in Fuzhou, China (26°04’ N, 119°18′E). As a representative subtropical evergreen broad-leaved forest ecosystem, Fuzhou National Forest Park harbours exceptionally rich regional flora. The overstory canopy is predominantly characterised by native broad-leaved species, prominently including the small-leaved banyan (*Ficus microcarpa*) and camphor tree (*Cinnamomum camphora*), interspersed with seasonal deciduous elements such as Chinese sweet gum (*Liquidambar formosana*). The complex understory consists of diverse shade-tolerant shrubs and herbaceous plants, such as shell ginger (*Alpinia zerumbet*) and false staghorn fern (*Dicranopteris pedata*). This naturally stratified forest stand not only ensures a high baseline of visual vegetation complexity, but also provides critical foraging and nesting habitats for local avifauna, serving as the ecological foundation for the therapeutic cross-modal inputs investigated in this study. Fuzhou National Forest Park and its surrounding urban interface were selected as the primary experimental domain to minimise confounding from differential spatial accessibility (Chen et al., 2024) and macro-meteorological variation, and to maintain alignment with the paradigm of Nghiem et al. (2021) (Figure 2). Three independent walking trails (~600 m to 1 km) were delineated to create a pronounced gradient in vegetation structure and species richness (Liu et al., 2025):

(1) High-biodiversity trail (HFP): Located within the park’s mature native forest interior; characterised by a complex forest stand structure comprising a continuous canopy, a dense understory of shrubs, and a rich herbaceous layer. This naturally stratified, multi-layered vegetation provides an optimal physical foundation for therapeutic visual stimuli and acoustic habitats (e.g., abundant avian vocalisations) with minimal anthropogenic noise.
(2) Low-biodiversity trail (MIP): Located in the park’s peripheral transitional zone, representing a structurally simplified monoculture urban plantation. It is dominated by uniformly manicured open lawns and formal planted borders lacking understory complexity, and therefore exhibits significantly lower fauna and flora diversity.
(3) Non-natural urban control (UHS): This site comprises a high-traffic-density road segment dominated by impervious paving and grey infrastructure. For experimental rigor, this condition is operationally defined as a “zero-biodiversity” control. While recognizing that urban hardscapes are rarely absolute biological voids, the perceived biodiversity at this location is effectively negligible relative to the forest trails. Consequently, it serves as a critical non-natural baseline to isolate and quantify the net restorative effects attributable to varying degrees of nature exposure.

### 2.2 Participants and recruitment

A total of 360 participants were recruited using a stratified sampling strategy, allocated equally across three age cohorts: young adults (18–35 yr., *n* = 120), middle-aged adults (36–59 yr., *n* = 120), and older adults (≥ 60 yr., *n* = 120). Recruitment channels included online social media platforms, offline community outreach, and random intercept at neutral public venues. All participants demonstrated intact visual and cognitive function and no ambulatory disability or severe psychological disorder. In order to detect main and interaction effects (Chen et al., 2026) at the current sample size, a within—subjects pre-post design was used, which effectively controls for individual differences and gives sufficient statistical power (1 − β > 0. 80) (Menzel et al., 2024).

To rigorously control for self-selection bias, participants within each age cohort were randomly assigned to one of the three environmental conditions (HFP, MIP, UHS) using a computer-generated randomization sequence prior to the field experiment. While a strict double-blind approach was not feasible given the visual nature of the intervention, participants were kept entirely naive to the environmental contrasts and the specific hypotheses regarding biodiversity until the final post-experiment debriefing. Data from any participants who failed to complete the full walking protocol (*n* = 0) were excluded prior to analysis.

### 2.3 Experimental design and procedure

The experimental field design employed was a rigorous quasi-experimental interventional design comprising pre-intervention, intervention, and post-intervention phases (Nghiem et al., 2021). Participants were informed only that they were participating in an “urban walking experience assessment”; the biodiversity focus of the study was not disclosed until debriefing. Data collection proceeded across multiple rainless days between October 15 and November 20, 2025, in batches of 10–20 participants. To control for diurnal variations in microclimate, sun angle, and natural soundscapes (e.g., bird activity peaks), all walking interventions were strictly scheduled during specific daily windows: 08:30–11:00 and 14:30–17:00. The protocol comprised three sequential phases (Korpilo et al., 2024):

T0 — Baseline: Participants provided informed consent and completed a T0 questionnaire capturing sociodemographic characteristics, habitual park visit frequency, and the first administration of the Positive and Negative Affect Schedule (PANAS).

T1—Walking intervention Participants began their walks at staggered intervals of one minute and maintained a self-selected pace for at least 20 min. No strict upper time limit was imposed. Participants were prohibited from conversing, listening to music, or using mobile devices during the walk (Korpilo et al., 2024). Research assistants recorded precise walking duration.

Since participants returned to the endpoint immediately, they were able to complete the T2 questionnaire battery on-site right after the T2 post-assessment.

### 2.4 Measurement instruments

The questionnaire designed for this study evaluates four main latent constructs: perceived biodiversity, perceived restorativeness, nature connectedness, and emotional states (positive and negative affect), along with a control variable for perceived crowding. To ensure methodological transparency and reproducibility, the complete measurement scales, construct dimensions, and specific item descriptions used in this study are detailed in Appendix A (Table A1). While the full sample (*N* = 360) is used for baseline equivalence and environmental main effect comparisons, the subsequent psychometric validation and MG-SEM are restricted to the natural trail subsample (*n* = 240). This is methodologically necessary because the urban control group had no forest exposure, making the “perceived biodiversity → PRS → PA” mediation model conceptually and mathematically inapplicable to them. To maintain transparency, instrument reliability is reported for both the full sample (*N* = 360) to confirm overall scale stability and the subsample (*n* = 240) to align with subsequent structural path analyses.

All psychometric tools showed outstanding reliability and validity in the context of the current large sample.

Affective well-being (T0 and T2) PANAS was used to evaluate immediate emotional states both prior to and following the intervention. Cronbach’s α for the entire sample (*N* = 360) exceeded 0.897 for both subscales at both time points, while the post-intervention α for the natural trail analytical subsample (*n* = 240) were 0.942 for PA and 0.923 for NA.

Perceived restorativeness (T2): The Perceived Restorativeness Scale (PRS) assessed environmental restorative potential across four dimensions (being away, coherence, compatibility, fascination); the scale exhibited high internal consistency across the entire recruited sample (*N* = 360, Cronbach’s α = 0.921) and remained highly reliable within the natural trail subsample (*n* = 240, Cronbach’s α = 0.862).

Perceived biodiversity was operationally defined and measured through three specific dimensions: (1) Visual Plant Complexity, assessing the perceived richness of layers, varied heights, and floral color diversity (e.g., “The plant community features rich layers with varied heights”); (2) Characteristic Natural Soundscape, evaluating the perceived diversity of birdsong and other natural sounds (e.g., “I can clearly hear the rich and diverse natural sounds of birds”); and (3) Faunal Abundance Estimation, reflecting the subjective appraisal of animal and insect inhabitants. This novel multisensory self-report scale demonstrates high structural validity (standardised loadings > 0.60) and excellent internal consistency across the entire sample (*N* = 360, Cronbach’s α = 0.962), as well as within the natural trail subsample (*n* = 240, Cronbach’s α = 0.926).

Nature relatedness (T2): The six-item Nature Relatedness Scale (NR-6) [12]; the Cronbach’s α was 0.911 for the entire sample (*N* = 360) and 0.928 for the natural trail subsample (*n* = 240).

Covariates: Actual walking duration (min; objectively recorded) and perceived crowding (ordered categorical variable).

### 2.5 Data analysis strategy

Analysis proceeded through three sequential stages: (1) Baseline equivalence testing via one-way ANOVA in SPSS 27.0.1; (2) Main effect estimation via repeated-measures ANOVA or paired-samples t-tests comparing affect change scores across environmental conditions; (3) MG-SEM path analysis (AMOS 24.0) restricted to participants from the two natural trails (*n* = 240), with age cohort as the grouping variable. T0 PA and NA were specified as direct predictors of T2 outcomes to isolate net affect change; NR-6, walking duration, and perceived crowding were entered as covariates. Cluster-robust standard errors (site ID as clustering variable) were applied throughout to correct for spatial non-independence.

To ensure the stability and precision of parameter estimates given the modest sub-sample sizes (*n* = 80 per group) in the multi-group analysis, a bias-corrected bootstrapping procedure with 5,000 resamples was employed. This non-parametric approach is a rigorous protocol in structural equation modelling (SEM) to address potential violations of multivariate normality and to generate reliable 95% confidence intervals for indirect effects. By iteratively resampling from the observed data, this method provides robust estimations of the mediation pathways and effectively manages small-sample instability, ensuring that the structural associations remain conservative and statistically sound without introducing systematic bias.

## 3 Results

### 3.1 Baseline characteristics and measurement invariance

The final sample spanned the full adult lifespan with marked heterogeneity in educational attainment and habitual park visitation (Table 1). Regarding potential confounds of site familiarity, chi-square tests revealed that park visit frequency did not differ significantly across the three age cohorts (*x*^2^ = 4.199, df = 6, *p* = 0.650). The distribution of daily, weekly, and rare visitors was statistically balanced across groups, suggesting that the reported age-cohort differences in the mediation model are not a reflection of differential place familiarity or residency patterns.

**Table 1:** Sociodemographic characteristics of the study sample (*N* = 360).

Variable	Category	*n*	%
Age group	Young adults (18–35 yr)	120	33.3
	Middle-aged adults (36–59 yr)	120	33.3
	Older adults (≥60 yr)	120	33.3
Sex	Male	182	50.6
	Female	178	49.4
Educational attainment	Junior high school or below	47	13.1
	Senior high school/vocational	102	28.3
	College/undergraduate	149	41.4
	Master’s degree or above	62	17.2
Park visit frequency	Rarely/never	98	27.2
	1–2 times per week	87	24.2
	3–5 times per week	93	25.8
	Daily or nearly daily	82	22.8

One-way ANOVA confirmed pre-intervention baseline equivalence: neither PA nor NA scores differed significantly across environmental conditions or age cohorts (all *p* > 0.05; Table 2).

**Table 2:** Baseline affective equivalence across environmental types and age cohorts.

Variable	Group	n	Baseline PA (M ± SD)	Baseline NA (M ± SD)
Environment	HFP	120	2.744 ± 0.519	2.233 ± 0.495
	MIP	120	2.747 ± 0.502	2.255 ± 0.499
	UHS	120	2.773 ± 0.479	2.235 ± 0.498
	F	—	0.118	0.074
	*p*	—	0.889	0.929
Age cohort	Young adults	120	2.739 ± 0.470	2.248 ± 0.478
	Middle-aged adults	120	2.766 ± 0.532	2.237 ± 0.550
	Older adults	120	2.758 ± 0.497	2.237 ± 0.460
	F	—	0.091	0.016
	*p*	—	0.913	0.984

Multi-group measurement invariance testing indicated configural and metric invariance (ΔCFI = 0.003 < 0.01; ΔRMSEA = 0.004 < 0.015), as shown in Table 3, supporting cross-group structural path comparison. Although the changes in the fit indices of the scalar invariance model exceeded the strict criteria (ΔCFI = 0.049, ΔRMSEA = 0.030), making it impossible to compare latent variable means across groups in a strict psychometric sense, in accordance with common practices in multi-group structural equation modeling research, the establishment of metric invariance is a prerequisite for cross-group comparison of structural path coefficients. This indicates that participants in different age groups exhibited consistency in their interpretation of the scale’s factor structure; intercept heterogeneity did not fundamentally affect the examination of structural relationships, thereby providing an acceptable measurement foundation for testing the age-gradient pattern of the mediating effect.

**Table 3:** Multi-group measurement invariance test results.

Model	χ^2^	df	χ^2^/df	CFI	RMSEA	Comparison	Δχ^2^	Δdf	ΔCFI	ΔRMSEA
M1: Configural	354.315	333	1.064	0.993	0.016	—	—	—	—	—
M2: Metric	367.268	355	1.035	0.996	0.012	M2 vs. M1	12.953	22	0.003 ✓	0.004 ✓
M3: Scalar	542.094	383	1.415	0.947	0.042	M3 vs. M2	187.779	50	0.049 ❌	0.030 ❌

✓ = within accepted threshold; ❌ = exceeds threshold.

### 3.2 Effects of perceived biodiversity and covariates

**Figure 3:**
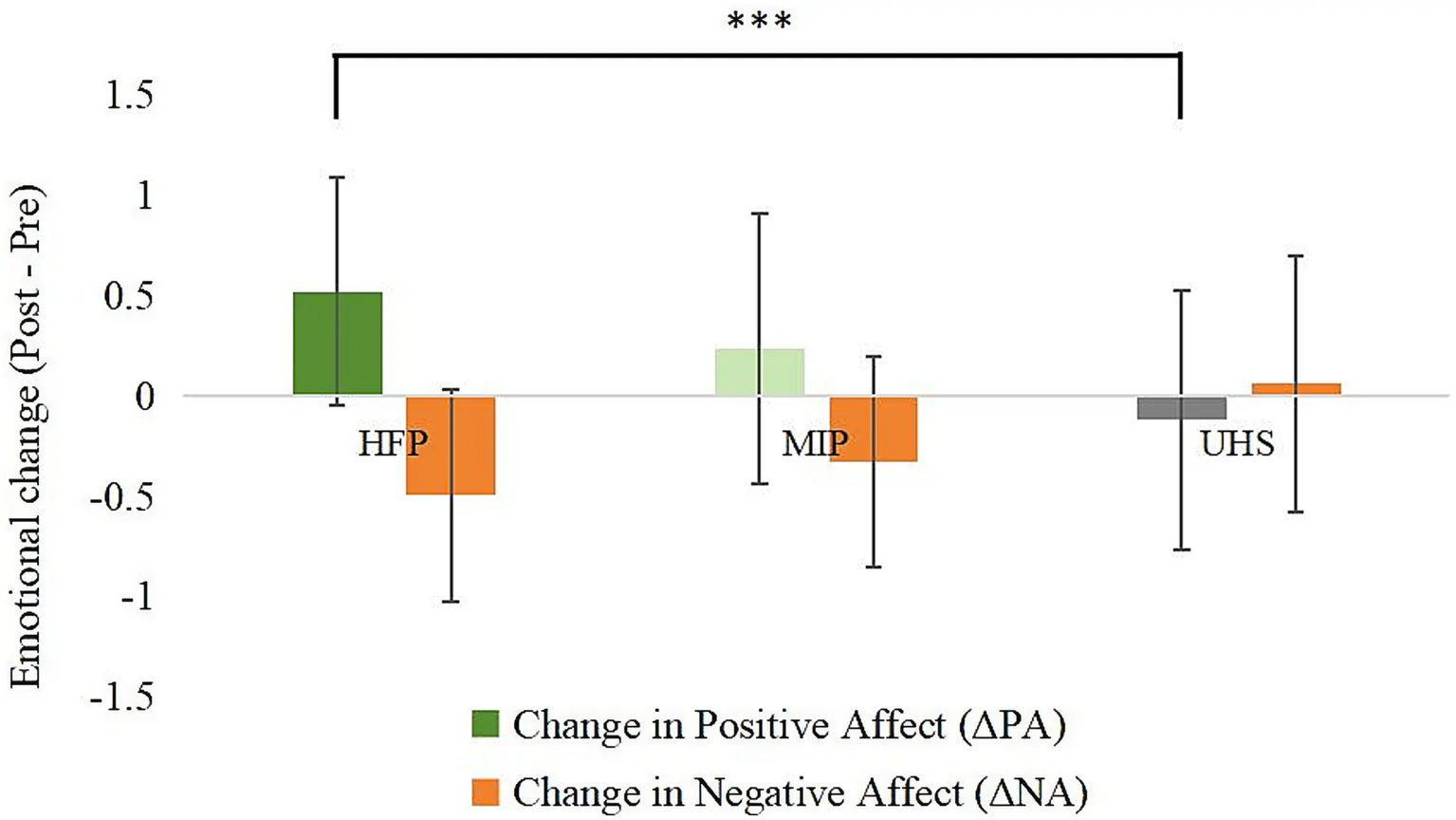
Pre-to-post changes in PA and NA across the three environmental typologies. Error bars represent standard error of the mean. *** *p* < 0.001; *n.s.* = not significant.

A repeated measures ANOVA indicated a significant interaction between environmental type and time regarding affective change. Both natural trails (HFP, MIP) produced significantly greater post-walk gains in positive affect (PA) than the UHS control (*p* < 0.001; Figure 3). No significant main effect was observed for negative affect (NA) (*p* > 0.05), consistent with a floor effect in baseline NA scores.

Prior to conducting the structural path analysis, the measurement model was rigorously evaluated (Table 4). All latent constructs demonstrated excellent internal consistency and composite reliability, with Cronbach’s α and Composite Reliability (CR) values all exceeding the stringent 0.85 threshold. Convergent validity was established, as the Average Variance Extracted (AVE) for all constructs comfortably exceeded the 0.50 benchmark. Furthermore, discriminant validity was strongly supported, given that the square root of the AVE for each construct (diagonal elements) was strictly greater than its corresponding off-diagonal bivariate correlations. The Pearson correlation matrix revealed theoretically coherent baseline relationships: perceived biodiversity, PRS, and NR-6 were all significantly and positively correlated with post-walk PA (all *p* < 0.01), thereby justifying their inclusion in the subsequent structural equation modelling.

**Table 4:** Reliability, validity, and Pearson correlation matrix (*n* = 240).

Variable	α	CR	AVE	1	2	3	4	5	6	7
1. Perceived biodiversity	0.926	0.908	0.665	0.815						
2. PRS	0.862	0.857	0.599	0.510 **	0.774					
3. NR-6	0.928	0.916	0.646	0.166 **	0.381 **	0.804				
4. PA (post)	0.942	—	—	0.272 **	0.483 **	0.205 **	—			
5. NA (post)	0.923	—	—	−0.186 **	−0.350 **	−0.156 *	−0.233 **	—		
6. Walking duration (min)	—	—	—	0.003	−0.061	−0.075	−0.065	0.061	—	
7. Perceived crowding	—	—	—	−0.050	−0.110	−0.022	0.017	0.047	0.038	—

Diagonal elements for latent variables = square root of AVE. ^ = observed variable (CR and AVE not applicable). α = Cronbach’s alpha; CR = composite reliability; AVE = average variance extracted. * *p* < 0.05, ** *p* < 0.01 (two-tailed).

Covariate pathway analyses within MG-SEM revealed several noteworthy patterns (Table 5). Walking duration showed a significant bivariate negative correlation with post-walk PA (r = −0.226, *p* < 0.01), likely reflecting biomechanical fatigue on hilly terrain; however, this effect was fully absorbed in the MG-SEM across all age groups (all *p* > 0.05). Perceived crowding negatively affected PRS (r = −0.216, *p* < 0.01) yet weakly and positively correlated with post-walk PA (r = 0.131, *p* < 0.05), suggesting a social safety signal effect in the wilderness-character park. Critically, the disruptive effect of crowding on PRS was significant for young adults (β = −0.283, *p* = 0.017) and middle-aged adults (β = −0.375, *p* < 0.001) but totally non-significant for older adults (β = −0.008, *p* = 0.896). NR-6 significantly predicted PRS across all groups yet exerted no significant direct effect on PA (all *p* > 0.05), indicating complete mediation by PRS.

**Table 5:** Standard path coefficients across age cohorts in the MG-SEM model.

Pathway	Young adults β	Middle-aged β	Older adults β
I. Primary direct effects			
Perceived biodiversity → PRS	0.196	0.782***	0.878***
PRS → PA	0.265	0.280	0.654***
II. Covariate direct effects			
NR-6 → PRS	0.370**	0.371***	0.408***
NR-6 → PA	0.020	0.005	0.088
Perceived crowdedness → PRS	−0.283*	−0.375***	−0.008
III. Indirect/mediation effect			
Perceived biodiversity → PRS → PA	0.052	0.219	0.574***

** *p* < 0.01, *** *p* < 0.001. Paths without significance markers were non-significant (*p* > 0.05).

### 3.3 Age-cohort differences in the mediation model

**Figure 4:**
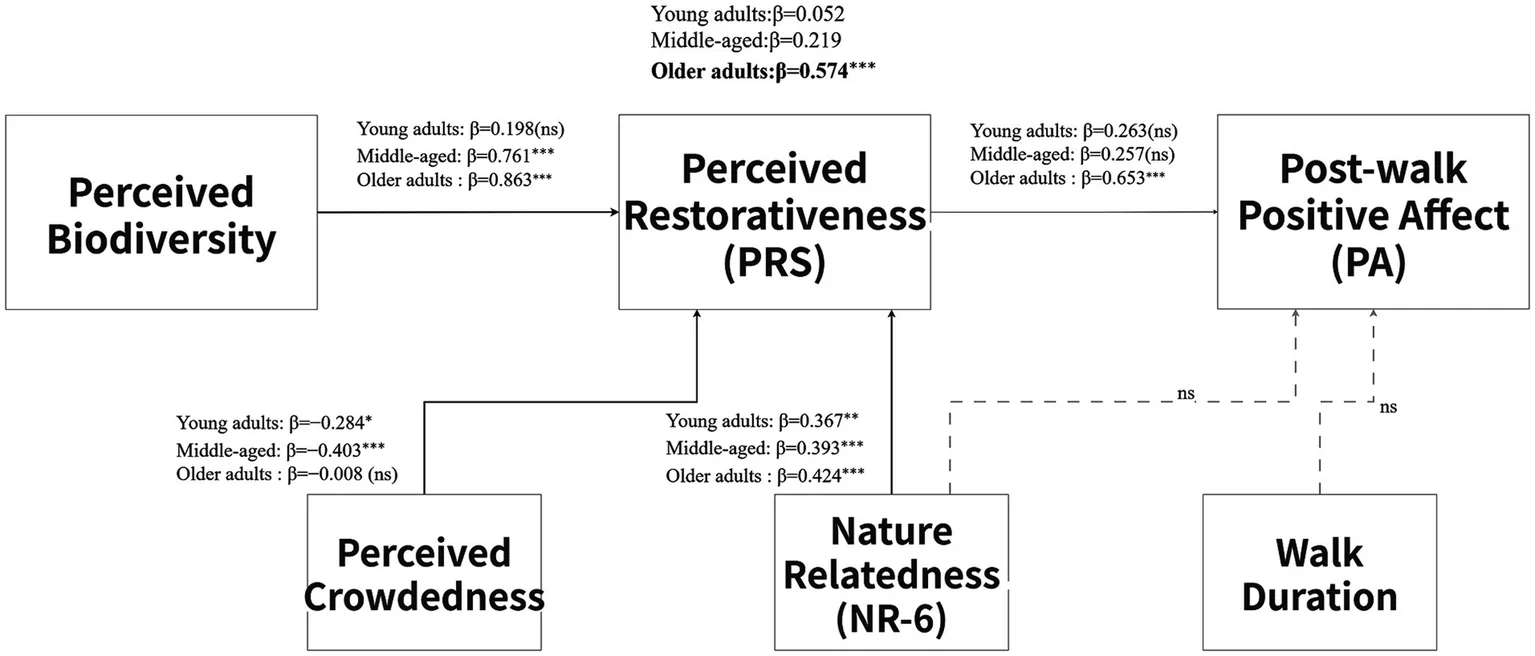
MG-SEM path diagrams illustrating age-cohort variations in the indirect association of perceived biodiversity on PA via PRS. Standardised path coefficients displayed for each age cohort. * *p* < 0.05, ** *p* < 0.01, *** *p* < 0.001. Standardized path coefficients without asterisks are non-significant (*p* > 0.05).

MG-SEM results revealed a pronounced age-cohort variations in the indirect association of perceived biodiversity on PA via PRS (Figure 4; Table 5). The indirect effect was non-significant for young adults (β = 0.052, *p* = 0.138), marginally non-significant for middle-aged adults (β = 0.219, *p* = 0.051), and highly significant for older adults (β = 0.574, *p* < 0.001). Pairwise multi-group critical ratio tests confirmed that the path coefficient for older adults was statistically significantly greater than for both young and middle-aged adults (*p* < 0.05). Among PRS dimensions, “fascination” and ‘coherence’ contributed disproportionately to affective improvements in older adults.

## 4 Discussion

### 4.1 Multisensory mechanisms of psychological restoration

The two key predictors identified — visual plant complexity and birdsong — provide a multisensory integrative lens for environmental psychology. This confirms that the health benefits of nature exposure are not merely derived from a homogenous ‘green’ backdrop, but are actively driven by specific ecological attributes. From the perspectives of SRT and evolutionary psychology, moderately complex plant communities furnish implicit biotic resource cues, while birdsong transmits an auditory signal of environmental safety and habitability. Consistent with ART, this cross-modal input — the soft geometrical contours of multi-layered vegetation and the animated biological rhythms of avian vocalisations — potently activates environmental fascination, gently engaging involuntary attention. Relative to unisensory stimulation, multisensory integrated input more efficiently interrupts negative ruminative cycles, facilitating the replenishment of depleted attentional resources and the offloading of cognitive burden (Chen et al., 2024).

### 4.2 Theoretical dialogue and novel contributions

Our findings strongly corroborate and substantially extend the conclusions of Nghiem et al. (2021), confirming that the perceived biodiversity → PRS → PA constitutes a robust associational pathway, and that nature exposure of at least 20 min is sufficient to trigger notable psychological benefits, though our design does not establish this duration as an absolute minimum threshold and is attenuated by perceived crowding (García-Martín et al., 2025). The negative impact of crowding aligns with a broad consensus in restorative landscape literature: high social density typically increases cognitive load and directly reduces the “being away” component of perceived restorativeness (Arnberger and Eder, 2015; García-Martín et al., 2025). Prior studies confirm that visitors seeking stress relief demonstrate a strong preference for lower crowding levels to facilitate psychological distancing from daily stressors (Arnberger and Eder, 2015). Building upon these foundations, the present study demonstrates two layers of indirect relationships: (1) at the environmental stimulus level, perceived biodiversity shows no significant direct association with PA, suggesting that multisensory features primarily correlate with outcomes through environmental fascination (Rozario et al., 2024); and (2) at the individual trait level, NR-6 exhibits no significant direct effect on PA, corroborating the complete mediation by PRS reported by Nghiem et al. (2021) and emphasising that even highly nature-affiliated individuals depend on the actual restorative potential (Jin et al., 2026) afforded by the physical environment.

The present study makes a clear, systematic advance beyond the existing literature along two well-defined dimensions. First, it goes beyond sample homogeneity by identifying distinct age-related variations in therapeutic benefit: the mediating efficacy of PRS is highly prominent and statistically significant in older adults (β = 0.574, *p* < 0.001) but remains non-significant in young adults (β = 0.052, *p* > 0.05). Thus it naturally extends ART by showing that age-related cognitive resource depletion makes older adults more environmentally dependent (Zhang et al., 2025) for psychological restoration. Second, it refines sensory granularity by translating macroscopic faunal perception into cross-modal visuoauditory integration (visual plant complexity + auditory birdsong), thereby offering more operationally concrete and compelling empirical support for urban green infrastructure planning and nature-based therapeutic design (Qi et al., 2022; Korpilo et al., 2024).

Crowding emerged as an informative covariate, revealing a notable age-related shift in environmental sensitivity. While the significant disruption of PRS among younger cohorts reinforces the “social escape” hypothesis prevalent in environmental psychology, our findings extend this by demonstrating that older adults exhibit a higher tolerance for social co-presence (García-Martín et al., 2025). Rather than indicating that older adults actively seek high-density social vitality, this statistical non-significance suggests that their restorative process is less fragile under social interference. This could be attributed to “social safety signals”—where the presence of others provides a sense of security for seniors in wilderness-character environments—thereby refining existing social-spatial theories through a lifespan lens (García-Martín et al., 2025). The underlying psychological drivers—whether related to safety reassurance or age-related shifts in spatial expectations—remain speculative and warrant targeted qualitative investigations in future research.

### 4.3 Limitations and future directions

Several methodological limitations warrant acknowledgement. First, regarding statistical interpretation, we have adopted a cautious and measured stance. While we employed cluster-robust standard errors (site ID) to account for intra-site nesting, we acknowledge the limitation of potential inter-site spatial spillovers which this method may not fully capture. Consequently, the reported pathways represent structural associations rather than definitive causal mechanisms. Future research should consider incorporating spatial weights matrices or employing factorial field experiments to more rigorously isolate these cross-modal effects and account for spatial non-independence. Second, our experimental design evaluated the natural environment as a composite whole without incorporating unisensory control conditions. Furthermore, although park visit frequency was statistically balanced across cohorts, we cannot entirely rule out the influence of nuanced place attachment or long-term residency, which may covary with age. Importantly, because our study employs a cross-sectional design involving only three discrete age groups rather than a continuous developmental sample, we cannot firmly establish a continuous age-ascending trajectory. The observed variations represent cohort-specific differences rather than a continuous aging gradient. Future longitudinal designs should explicitly incorporate residency status as a covariate and utilize a continuous age-sampling spectrum to clarify these lifespan trajectories to isolate the effects of environmental novelty versus long-term familiarity on psychological restoration. Consequently, while we theorize the benefits of “visuoauditory integration,” our design cannot statistically isolate whether these sensory inputs acted synergistically or merely additively. Future factorial field experiments are required to rigorously test true cross-modal synergistic effects. Finally, data collection was confined to a single seasonal window, potentially obscuring phenological dynamics. Future research should incorporate longitudinal cohorts and wearable neurophysiological monitoring to capture dynamic, causal responses.

## 5 Conclusion

Utilizing a large-sample quasi-experimental design and MG-SEM, this study is the first to uncover critical predictive relationships between multisensory interactions in UGS—particularly visual plant complexity and birdsong—and public affective well-being. It also demonstrates that these relationships show significant age-cohort variations in their mediating mechanisms. Our findings dismantle the conventional therapeutic landscape paradigm’s over-reliance on single visual greenness metrics, foreground the centrality of cross-modal visuoauditory integration in activating restorative potential, and fill a significant gap regarding multisensory perception in ageing populations.

Since the study provides a precise framework for age-inclusive urban public space planning, it is imperative for future landscape design practices to pivot from merely expanding macro-level canopy cover to enhancing site-level microecological diversity. By employing biodiversity-oriented design and mixed-species planting strategies that create visible plant stratification and actively restoring avian habitats (generating therapeutic natural soundscapes), urban planners can maximise the restorative potential of urban green spaces. Such environmental interventions will serve as a crucial pathway to building genuinely age-inclusive therapeutic cities and maximising public health benefits.

## Data Availability

The raw data supporting the conclusions of this article will be made available by the authors, without undue reservation.

## Supplementary material

The Supplementary material can be found online at: https://www.frontiersin.org/articles/10.3389/fpsyg.2026.1907289/full#supplementary-material
