# Supplementary material for: Restorative effects of urban biodiversity: a study based on age differences using multigroup structural equation modeling
Source: Front Psychol. 2026 Aug 28;17:1907289. doi: 10.3389/fpsyg.2026.1907289 (PMC13561792; doi:10.3389/fpsyg.2026.1907289)
Supplement: Supplementary file 1 [file Table_1.docx]

**Appendix A Complete Measurement Scales and Items**

Appendix A.1 Constructs, Dimensions, and Item Descriptions used in the questionnaire.

| **Latent Variable (Construct)** | **Dimension** | **Item Code** | **Item Text** | **Scale / Anchors** |
| --- | --- | --- | --- | --- |
| Perceived Biodiversity | Visual Plant Complexity | VP1 | The plant community along the trail features rich layers with varied heights and spatial arrangements. | 5-point Likert scale |
| (Self-developed/Adapted for this study) |  |  |  |  |
|  |  |  |  | (1 = Strongly disagree, 5 = Strongly agree) |
|  |  | VP2 | The plants along the trail display a rich variety of colors (e.g., varying shades of green leaves, diverse floral colors). | Same as above |
|  | Characteristic Natural Soundscape | NS1 | During the walk, I can clearly hear the natural sounds of birds. | Same as above |
|  |  | NS2 | The natural sounds along the trail (e.g., bird songs, insect calls, wind rustling through leaves) are rich and diverse. | Same as above |
|  | Faunal Abundance Estimation | FA1 | I perceive that there is a high abundance of various natural animals or insects inhabiting the area around this trail. | Same as above |
| Perceived Restorativeness | Being Away | PRS1 | Being here helps me feel away from my daily routines and stress, allowing me to take a breath. | 5-point Likert scale |
| (Based on PRS-short version) |  |  |  |  |
|  |  |  |  | (1 = Strongly disagree, 5 = Strongly agree) |
|  | Fascination | PRS2 | The natural environment here is engaging and holds my attention effortlessly. | Same as above |
|  | Coherence | PRS3 | The natural elements here harmonize well, forming a coherent overall environment. | Same as above |
|  | Compatibility | PRS4 | This environment fits my personal needs, and I can do what I want to do here. | Same as above |
| Nature Connectedness | Individual-Nature Relationship | NC1 | I feel a deep and strong connection with the natural world. | 1-5 Likert scale |
| (Based on NR-6) |  |  |  |  |
|  |  |  |  | (Match your actual scale) |
|  |  | NC2 | I always think of myself as a part of nature, not separate from it. | Same as above |
|  |  | NC3 | Even in the city, I often notice the wildlife and plants around me. | Same as above |
|  |  | NC4 | I am clearly aware that my personal actions impact the health of the natural environment. | Same as above |
|  |  | NC5 | I believe all living things have intrinsic value, regardless of their usefulness to humans. | Same as above |
|  |  | NC6 | I prefer to spend time in natural environments rather than indoors or in urban buildings. | Same as above |
| Perceived Crowding | Social Environment Perception | CR1 | The number of people on the trail made me feel crowded or interfered with my experience. | 5-point Likert scale |
| Positive and Negative Affect | Positive Affect (PA) | PA1 - PA10 | Interested, Excited, Strong, Enthusiastic, Proud, Alert, Inspired, Determined, Attentive, Active. | 5-point Likert scale |
| (Based on PANAS) |  |  |  |  |
|  |  |  |  | (1 = Very slightly or not at all, 5 = Extremely) |
|  | Negative Affect (NA) | NA1 - NA10 | Distressed, Hostile, Irritable, Guilty, Scared, Jittery, Nervous, Afraid, Ashamed, Upset. | Same as above |
